# Simultaneous detection and differentiation of three genotypes of Brassica yellows virus by multiplex reverse transcription-polymerase chain reaction

**DOI:** 10.1186/s12985-016-0647-7

**Published:** 2016-11-22

**Authors:** Xiaoyan Zhang, Yanmei Peng, Ying Wang, Zongying Zhang, Dawei Li, Jialin Yu, Chenggui Han

**Affiliations:** 1 State Key Laboratory for Agrobiotechnology and Ministry of Agriculture Key Laboratory for Plant Pathology, China Agricultural University, Beijing, China; 2 State Key Laboratory of Agrobiotechnology, College of Biological Sciences, China Agricultural University, Beijing, China

**Keywords:** Brassica yellows virus, Genotype, Multiplex RT-PCR, Detection, Crucifer crops

## Abstract

**Background:**

Brassica yellows virus (BrYV), proposed to be a new polerovirus species, three distinct genotypes (BrYV-A, BrYV-B and BrYV-C) have been described. This study was to develop a simple, rapid, sensitive, cost-effective method for simultaneous detection and differentiation of three genotypes of BrYV.

**Results:**

In this study, a multiplex reverse transcription-polymerase chain reaction (mRT-PCR) was developed for simultaneous detection and differentiation of the three genotypes of BrYV. The three genotypes of BrYV and *Tunip yellows virus* (TuYV) could be differentiated simultaneously using six optimized specific oligonucleotide primers, including one universal primer for detecting BrYV, three BrYV genotype-specific primers, and a pair of primers for specific detection of TuYV. Primers were designed from conserved regions of each virus and their specificity was confirmed by sequencing PCR products. The mRT-PCR products were 278 bp for BrYV-A, 674 bp for BrYV-B, 505 bp for BrYV-C, and 205 bp for TuYV. Amplification of three target genotypes was optimized by increasing the PCR annealing temperatures to 62 °C. One to three fragments specific for the virus genotypes were simultaneously amplified from infected samples and identified by their specific molecular sizes in agarose gel electrophoresis. No specific products could be amplified from cDNAs of other viruses which could infect crucifer crops. Detection limits of the plasmids for multiplex PCR were 100 fg for BrYV-A and BrYV-B, 10 pg for BrYV-C, and 1 pg for TuYV, respectively. The mRT-PCR was applied successfully for detection of three BrYV genotypes from field samples collected in China.

**Conclusions:**

The simple, rapid, sensitive, and cost-effective mRT-PCR was developed successfully for detection and differentiation of the three genotypes of BrYV.

## Background

Brassica yellows virus is a newly identified species in the genus of *Polerovirus*, which was closely related to, but distinct from *Tunip yellows virus* (TuYV) in terms of P0, P3, P4 and P5 gene sequences. Brassica yellows virus (BrYV) is an aphid-transmitted and phloem-limited virus as other poleroviruses do (unpublished data). BrYV is distributed widely in mainland China, as well as in South Korea and Japan [1–3]. BrYV can infect nine cruciferous plant species including cabbage (*Brassica oleracea var. capitata*), Chinese cabbage (*B. pekinensis*), cauliflower (*B. oleracea var. botrytis*), flowering Chinese cabbage (*B. chinensis*), leaf mustard (*B. juncea*), oilseed rape (*B. napus*), rutabaga (*B. napobrassica*), white glabrous mustard (*B. alboglabra*) and radish (*Raphanus sativus var. oleifera*). It can cause yellowing and leaf malformation or mottling symptoms on cruciferous crops [1, 2].

The BrYVs were divided into three genotypes (BrYV -A, -B, and -C) according to sequence comparisons and phylogenetic analysis. BrYV has a single-stranded, positive-sense RNA genome containing six open reading frames (ORFs), and the three genotypes of BrYV have greater divergence in ORFs 0, 1 and 2 than in ORFs 3, 4 and 5 [1, 4]. For convenience of the virus research, the full-length infectious cDNA clones of the three genotypes were constructed under control of the cauliflower mosaic virus 35S promoter. The infectivity of the cDNA clones of the three genotypes in *Nicotiana benthamiana* was further confirmed by reverse transcription-PCR (RT-PCR), western blot and northern hybridization [5].

RT-PCR with universal primers (PoconF/ PococpR) was used for the amplification of a 1.4 kb band for poleroviruses [1, 6]. As to BrYV, it can be further determined by western blot and northern blot detection [5]. However, these methods could not distinguish the three genotypes of BrYV at a time. Multiplex RT-PCR can be used for simultaneously detection of several viruses in one reaction, and it provides a quick, efficient, reliable and economical way for detection of plant viruses and strains, especially in field samples [7–31].

The aim of this study was to develop a simple, rapid, sensitive, cost-effective method for simultaneous detection and differentiation of three genotypes of BrYV and TuYV. This multiplex RT-PCR assay developed here can act as a universal diagnostic tool for detection and epidemiological investigation of the three genotypes of BrYV.

## Methods

### Plant material and recombinant plasmids

*N. benthamiana* leaf tissues infected with BrYV-A, -B, C were used to standardize the multiplex RT-PCR. Each of these three genotypes infecting *N. benthamiana* was identified by RT-PCR and sequencing. Plasmids containing full-length genomic sequences of BrYV-A (Accession No. NC016038), BrYV-B (Accession No.HQ388351), BrYV-C (Accession No. KF015269), *Turnip mosaic virus*(TuMV) (Accession No. AF169561.2), *Cucumber mosaic virus* (CMV Fny strain) (Accession No. NC002034, NC002035, NC001440), *Cucurbit aphid-borne yellows virus* (CABYV) (Accession No. HQ439023), *Beet western yellows virus* Inner Mongolia isolate (BWYV-IM) (Accession No. EU636991), and plasmids containing partial genomic sequences of TuYV were used to test specificity of the multiplex PCR in this study. The plasmid pMD19-TuYV-P0 contained the complete sequence of the ORF0 of TuYV (Accession No. NC003743) and the plasmid pMD19-56#-1 contained the 4959–5163 nt sequence of TuYV which shared 96.4% nucleotide sequence identity with TuYV isolate Anhui (Accession No. KR706247.1).

### RNA extraction

Total RNA from 0.1 g of leaf tissue from infected plants was prepared by SDS-phenol/chloroform extraction [32]. 600 ul of phenol: chloroform and 630 ul of extraction buffer (20 mM Tris–HCl, pH 7.8, 1% sodium dodecyl sulfate, 200 mM sodium chloride, and 5 mM EDTA) were added with continuous homogenizing. The thawed mixture was extracted two times with phenol: chloroform and separated by centrifugation. Total RNA in the supernatant was precipitated by equal volume of 4 M lithium chloride. Then it was washed two times with chilled 70% ethanol and one time with chilled 100% ethanol. The RNA was eluted in a final volume of 40 μl of diethylpyrocarbonate-treated (DEPC) water and stored at −20 °C for the following protocols.

### Reverse transcription and PCR

The revere transcription (RT) reaction was carried out using TaKaRa RNA PCR Kit (M-MLV) Ver. 3.0 (Takara, Dalian, China) according to the manufacturer’s protocol. The reaction was performed in a 20 μl PCR master mixture consisting of 4 μl 5 × RT Buffer, 1 μl BrY761R (10 uM), 1 μl TuY5163R (10 uM), 9 μl RNase Free ddH_2_O, 1 μl dNTP Mixture (each 2.5 mM), 0.5 μl RNase Inhibitor (40 U/μl), 0.5 μl MLV Reverse Transcriptase (200 U/μl) and 3 μl plant total RNA (~3000 ng). The RT reaction was incubated for 90 min at 37 °C. The products were stored at −20 °C for uniplex RT-PCR and multiplex PCR reactions. Uniplex PCR for BrYV was carried out in a 25 μl mixture containing 12.5 μl 2 × TSINGKE Master Mix (blue) (Tsingke Biological Technology Company, Beijing, China), 2 μl cDNA template, 0.5 μl of specific primers (10 μM) (Table 1), and 9.5 μl ddH_2_O. Multiplex PCR reactions was carried out in a 25 μl mixture containing 12.5 μl 2 × TSINGKE Master Mix (blue), 2 μl cDNA template, 0.5 μl of the six primers (10 μM) for each (Table 1), and 7.5 μl ddH_2_O.

**Table 1:** List of primers used for developing multiplex RT-PCR

Primer	Primer sequence (5′ to 3′)	Positions
BrYA484F	5′- TACTTGGACTAGAGATGCTGAAAG-3′	BrYV-A 484–507 nt
BrYB88F	5′- CCTCCACCCAAAACAAGTAT-3′	BrYV-B 88–107 nt
BrYC257F	5′- CGAGTTTCCGTACTTGTTG-3′	BrYV-C 257–275 nt
BrY761R	5′- AGACCGAAGAGCTGAAAAGG-3′	BrYV-A 742–761 nt
TuY4959F	5′- AAGAGGCTTGCCCCTTCCTG-3′	TuYV 4959–4978 nt
TuY5163R	5′- AACCAAATCCGGTGTTGGAT-3′	TuYV 5144–5163 nt

Amplified products (10 μl each) were electrophoresed in 1.5% agarose gels and stained with ethidium bromide to confirm the expected size of the fragments.

### Cloning and sequencing

Purified PCR products, amplified with primers BrYA484F/ BrY761R, BrYB88F/ BrY761R, BrYC257F/ BrY761R from RT reaction products were inserted into pMD19-T and then transformed into competent cells of *Escherichia coli* MC1022. Recombinant clones were sequenced by Institute of Crop Science of CAAS (Beijing, China).

### Primer design and optimization of annealing temperatures

The full-length genomic sequences of BrYV and TuYV (BrYV-A^BJ^, NC016038; BrYV-A^JS^, HQ388350; BrYV-B^BJ^, HQ388349; BrYV-B^JS^, HQ388351; BrYV-C^R^, JN015068 and BrYV-C^C^, KF015269; TuYV, Nc003743; TuYV isolate Anhui, KR706247.1) were obtained from GenBank. The sequences of the designed primers used in this study are listed in Table 1. Gradient PCR was performed using different temperatures that were set randomly from 60 °C to 66 °C (60.0 °C; 60.6 °C; 61.4 °C; 62.4 °C; 63.8 °C; 64.9 °C; 65.5 °C; 66.0 °C) by the PCR machine.

### Sensitivity of multiplex RT-PCR

The sensitivity was considered as the lowest concentration of viral RNA giving a strong positive signal in mRT-PCR. To determine this threshold, ten-fold serially diluted DNA templates of the three genotypes of BrYV and TuYV were tested. The DNA samples range in quantity from 1 fg to 10 ng and the assay was carried out as described above.

### Detection of virus genotypes in field samples by multiplex RT-PCR

The samples showing symptoms such as yellowing and curl on leaves were collected from different fields in Haidian District of Beijing City. These samples were simultaneously used to test BrYV three genotypes by uniplex RT-PCR and multiplex RT-PCR.

## Results

### Primer design and optimization of multiplex RT-PCR

**Fig. 1:**
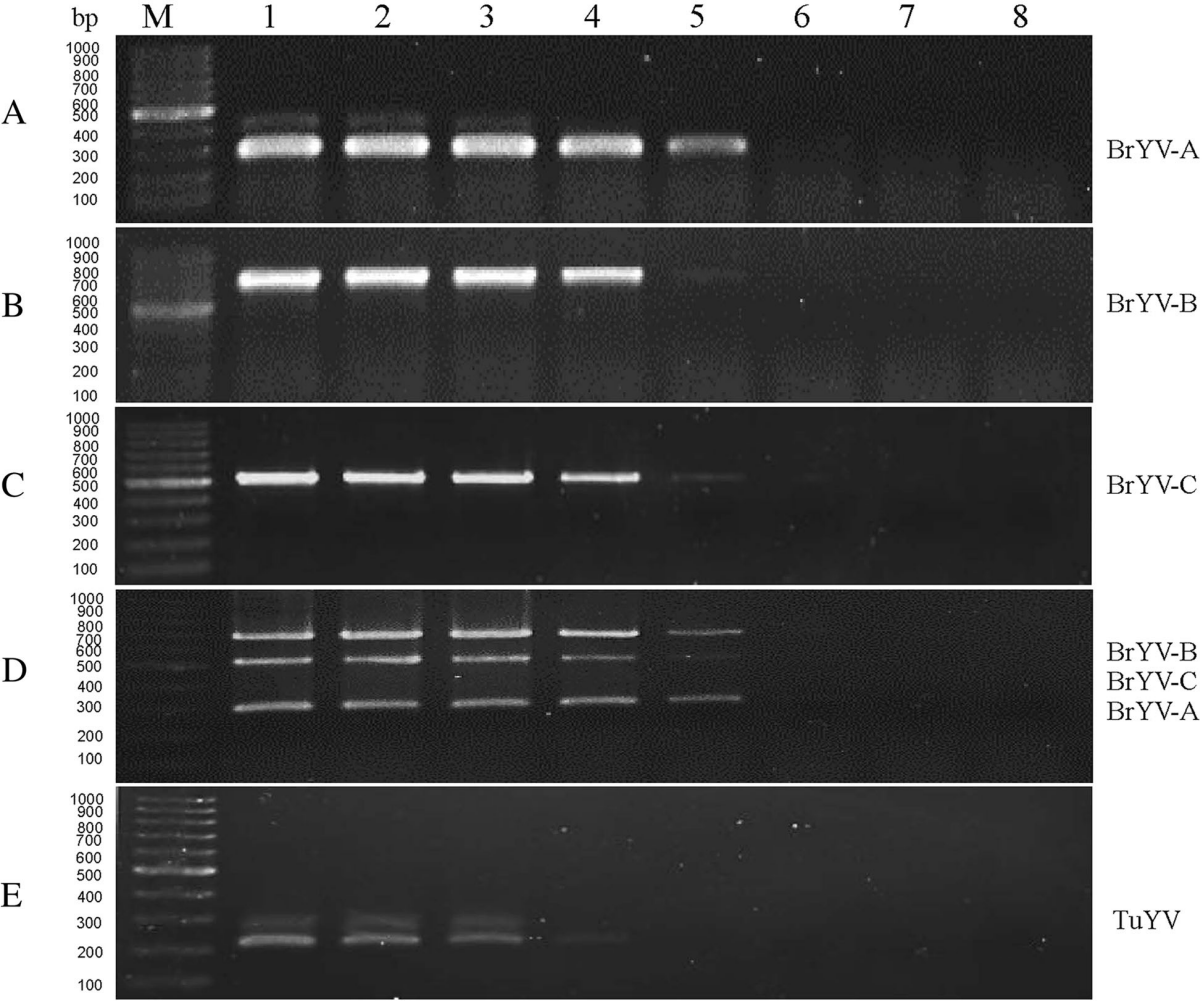
Optimization of the annealing temperature for uniplex RT-PCR of (**a**) BrYV-A (278 bp); (**b**) BrYV-B (674 bp); (**c**) BrYV-C (505 bp); (**d**) multiplex RT-PCR assay and (**e**) TuYV (205 bp) . (**a**, **b**, **c**, **e**), uniplex RT-PCR; (**d**), multiplex RT-PCR. Lane M: 100 bp DNA Marker; Lanes 1–8, 60.0 °C, 60.6 °C, 61.4 °C, 62.4 °C, 63.8 °C, 64.9 °C, 65.5 °C, 66.0 °C

Analysis of the complete genome sequences of six BrYV isolates showed that they represent three genotypes (BrYV-A, -B and -C). The three genotypes had more divergent in ORF0 which shared 90.4-92.5% nucleotide sequence identity. Four primers BrYA484F/ BrYB88F/ BrYC257F/ BrY761R were newly designed in the conserved regions within ORF0 of each genotype to allow for mRT-PCR. The three genotypes of BrYV were closely related to, but distinct from, TuYV in terms of P0, P3, P4 and P5 gene sequences [1, 4]. In order to detect TuYV at the same time, a pair of primers TuY4959F/TuY5163R in the conserved regions within ORF5 were designed (Table 1). The expected gene size of amplification were 278 bp for BrYV-A, 674 bp for BrYV-B, 505 bp for BrYV-C, and 205 bp for TuYV. A gradient RT-PCR was then performed to determine the optimal annealing temperature for uniplex RT-PCR and multiplex RT-PCR. Annealing temperatures between 60 °C and 66 °C were tested in order to optimize amplification by the gradient PCR machine (Fig. 1). According to efficiency and specificity of amplification for the targeted virus genotypes as detected by agarose gel electrophoresis, the optimal annealing temperature for mRT-PCR was determined to be 62 °C. The optimized cycle protocol of the multiplex RT-PCR was: 10 min at 95 °C; 32 cycles of at 30 s at 94 °C, 30 s at 62 °C; 1 min at 72 °C and a final extension at 72 °C for 10 min. Reaction products were electrophoresed in 1.5% agarose gels and stained with ethidium bromide.

### Sensitivity of multiplex RT-PCR

**Fig. 2:**
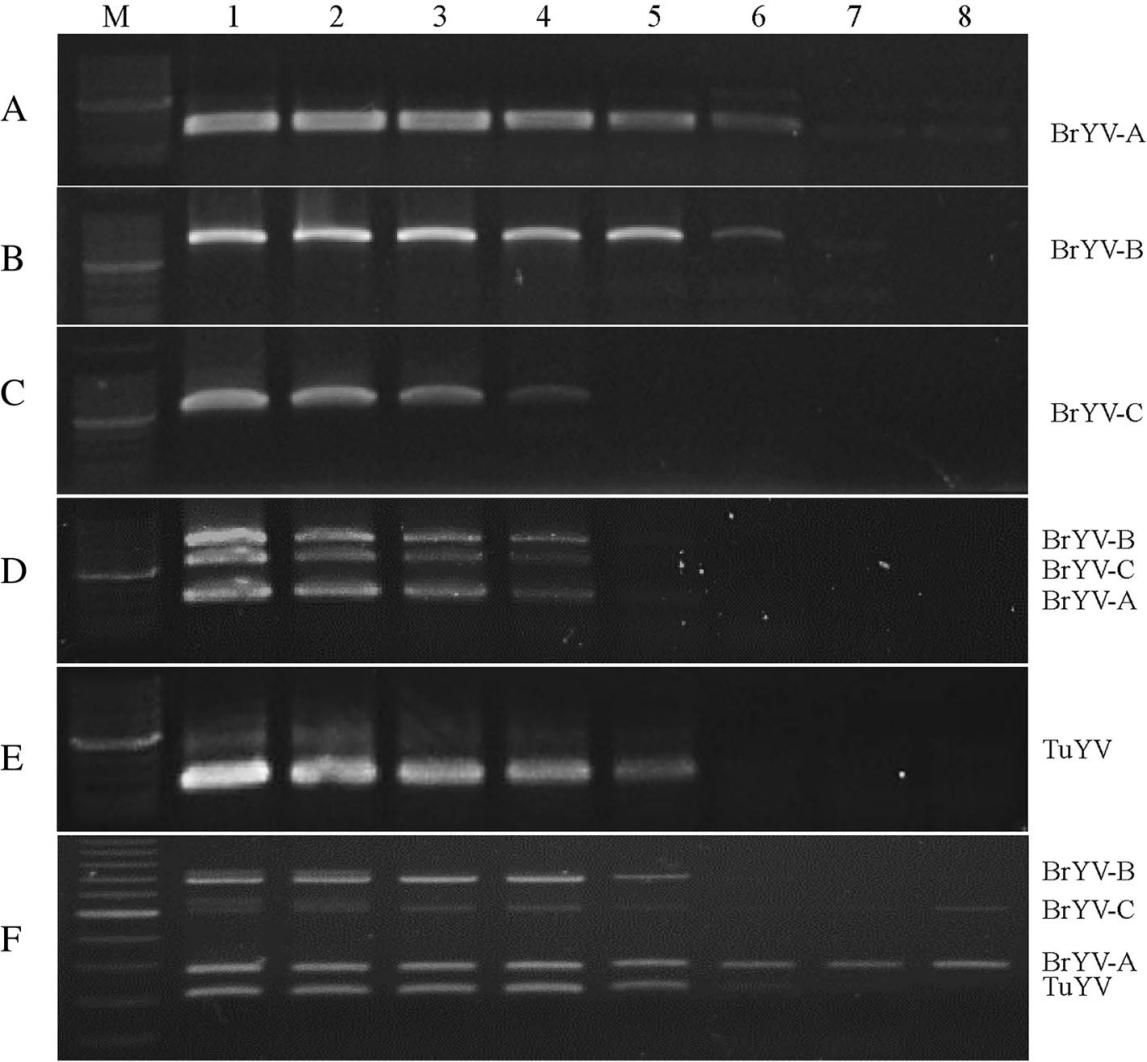
Detection limit of multiplex RT-PCR using ten-fold serial dilutions of individually plasmids. (**a**, **b**, **c**, **e**), uniplex PCR; (**d**, **f**), multiplex PCR. Lane M, 100 bp DNA marker. Lanes 1–8, 10 ng, 1 ng, 100 pg, 10 pg, 1 pg, 100 fg, 10 fg, and 1 fg of plasmids of different BrYV genotypes. (**e**), pMD19-TuYV-P0 and pMD19-56#-1 were used for dectection of TuYV

To compare the sensitivity of multiplex RT-PCR versus uniplex RT-PCR, 10-fold serial dilutions of the purified plasmids pCaBrA, pCaBrB, pCaBrC, pMD19-TuYV-P0, and pMD19-56#-1 were used simultaneously as templates in uniplex RT-PCR and multiplex RT-PCR reactions. The results showed that detection limits of the DNA quantity for multiplex RT-PCR were 100 fg for BrYV-A and BrYV-B, 10 pg for BrYV-C, and 1 pg for TuYV, respectively, if the concentration of the plasmids was similar (Fig. 2).

### Specificity of multiplex RT-PCR

**Fig. 3:**
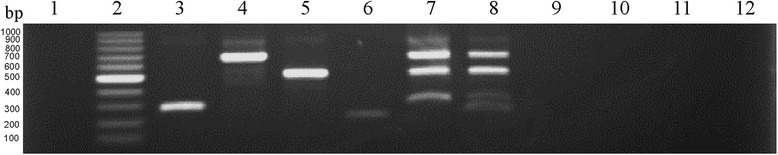
Specificity of the multiplex RT-PCR for detection of BrYV-A, BrYV-B, BrYV-C, and TuYV. Lanes 1 show healthy *Nicotiana benthamiana .* Lane 2, 100 bp DNA marker. Lanes 3–5 show amplification of individual BrYV genotype products. Lane 6 shows pMD19-TuYV-P0 and pMD19-56#-1. Lane 7 shows BrYV-A, BrYV-B and BrYV-C co-infected *N. benthamiana.* Lane 8 shows the BrYV-A, BrYV-B and BrYV-C co-infected *N. benthamiana* plus pMD19-TuYV-P0 and pMD19-56#-1*.* Lanes 9–12 show the plasmids containing genome sequences of *Turnip mosaic virus* (TuMV), *Cucumber mosaic virus* (CMV), *Cucurbit aphid-borne yellows virus* (CABYV) and *Beet western yellows virus* Inner Mongolia isolate (BWYV-IM) infectious cDNA clones

Using individual genotype RNAs and co-infected RNAs with three genotypes, the more clear and specific PCR products were detected in samples, respectively. Plasmids pMD19-TuYV-P0 and pMD19-56#-1 containing partial genomic cDNA sequences of TuYV were detected and gave a specific band. Plasmids containing cDNA sequences from two other crucifer-infecting viruses *Turnip mosaic virus* (TuMV), *Cucumber mosaic virus* (CMV), and two other poleroviruses *Cucurbit aphid-borne yellows virus* (CABYV) and *Beet western yellows virus* (BWYV) were used to test the specificity of the multiplex PCR, and gave no signal (Fig. 3).

### Detection of the three BrYV genotypes in field samples

**Fig. 4:**
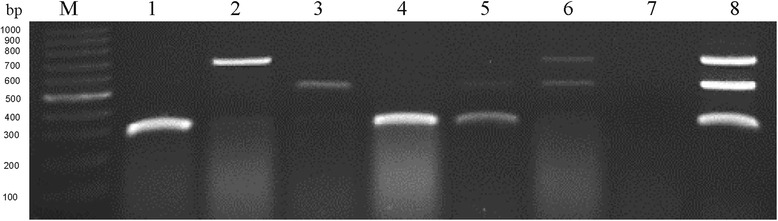
Agarose gel electrophoresis of 6 field samples collected from Haidian District of Beijing City by multiplex RT-PCR. Lanes 1–6, different crucifer leaf tissue samples collected from different fields in Haidian District of Beijing City, Lane 1, *Raphanus sativus L.*; Lane 2, *Brassica oleracea var. acephala f.tricolor*; Lane 3, *Brassica oleracea L. var. botrytis L.*; Lane 4, *Brassica rapa pekinensis*; Lane 5, *Brassica oleracea L. var. Capitata f. rubra DC.*; Lane 6, *Brassica oleracea L. var. caulorapa DC.*; Lane 7, negative control, the healthy crucifer plant sample; Lane 8, positive control, BrYV-A, -B and -C co-infected *Nicotiana benthamiana*

To evaluate the feasibility of multiplex RT-PCR for the diagnosis of field samples, 6 crucifer leaf tissue samples collected from different fields in Haidian District of Beijing City were used to test and standardize the mRT-PCR. One or two genotypes of BrYV could be detected by the mRT-PCR. The total RNA of BrYV-A, -B and -C agro-infected *N. benthamiana* served as positive control and it could produce three distinct fragments, 278 bp, 674 bp and 505 bp, respectively (Fig. 4). The healthy plant samples served as negative control gave no signal as expected. Following gel electrophoresis, the bands of PCR products were clear and there were no discernible differences in the size of the virus amplicons obtained from the different field samples indicating that the multiplex PCR assay could be used to detect the three genotypes of BrYV from a wide range of field areas. And the detection results were further confirmed by sequencing PCR products. With the high sensitivity and specificity of the mRT-PCR method, it is easy to detect and differentiate the three genotypes of BrYV.

## Discussion

The multiplex RT-PCR assay has progressed to become a convenient, rapid, and cost-effective tool for identification of different pathogens [33, 34]. Particularly, this technique has been used successfully for simultaneous detection and differentiation of the five seedborne legume viruses [35], three ilarviruses affecting stone fruit trees [27], five potato viruses and a viroid [8], six citrus viroids and apple stem grooving virus from citrus plants [36], four viruses and *Pseudomonas savastanoi* pv. savastanoi in olive trees [10], five tospoviruses in ornamental crops [28], eight stone fruit viruses [37], nine grapevine viruses [12], three beet poleroviruses in sugarbeet and aphids [38, 39], eight wheat viruses [13], nine crinivirus infecting vegetable and small fruit crops [15], four viruses infecting cassava [18], five tobacco viruses [19], three cucurbit-infecting poleroviruses [30], seven main tomato-infecting viruses [20], three rice viruses [21], three lily-infecting viruses [22], four cherry viruses [23], four viruses in peach [24], three viruses in pear plants [40], five viruses and two viriods infecting chrysanthemum [41], three viruses infecting papaya [42], three orchid viruses [25], five fabaviruses [31] and so on.

The multiplex RT-PCR method was also used for simultaneous detection and differentiation of virus subgroups, strains, genotypes or isolates. Multiplex RT-PCR assay has been developed successfully to detect and differentiate closely related but biologically distinct cherry isolates of *Prunus necrotic ringspot virus* [7], each of the major *Potato virus Y* strains and strain mixtures [43–46]. CMV subgroups and tobamoviruses infecting tomato [17], genotypes A and B of *Beet necrotic yellow vein virus* [11], five genotypes of *Citrus tristeza virus* from 29 citrus-growing countries [29]. There are three BrYV genotypes (BrYV -A, -B, and -C) based on sequence comparisons and phylogenetic analysis [1, 4], however, the pathogenicity, distribution and host range of the three BrYV genotypes remained to be determined. Therefore, the discrimination and specific detection of the three BrYV genotypes is essential in order to investigate the role these genotypes play in the epidemiology of the virus associated disease.

In this study, we developed a rapid, sensitive method for simultaneous detection of the three genotypes of BrYV. Generally, primer pairs are important factors affecting the efficiency and specificity of a multiplex RT-PCR. Therefore, four primer pairs were designed according to the conserved region in the P0 gene of three genotypes of BrYV and the conserved region in the P5 gene of TuYV. Amplification of three target genotypes was optimized by increasing the PCR annealing temperature to 62 °C, which produced amplicons of the appropriate size for each genotype target without extraneous banding (Fig. 1). There are some other viruses that can infect cruciferous crops. TuMV is one of the most devastating threats to the cruciferous crops [26, 47, 48]. CMV has extremely wide host range, and is one of the main pathogens of cruciferous crops [49]. Amplification of plasmids containing cDNA sequences from TuMV and CMV giving no signal indicated the specificity of the multiplex PCR. In the future, a multiplex RT-PCR assay should be developed for simultaneous detection and differentiation of BrYV three genotypes, TuYV, TuMV and CMV in cruciferous crops.

## Conclusions

In conclusion, a multiplex RT-PCR assay was developed to detect the three genotypes of BrYV (BrYV-A, -B and -C) from infected cruciferous crops. The multiplex PCR assay developed here is a rapid, reliable, sensitive and cost-effective diagnostic method. It can simultaneously detect and differentiate these three genotypes of BrYV and TuYV in infected leaf tissue across a wide range of field samples in one single reaction. However, the assay presented here accounts for known sequence variants of the three genotypes but cannot rule out the presence of variants yet to be sequenced. With this method, it would be useful for discerning the differences among the distribution, host range, pathogenicity, virus–plant-vector interactions and better understanding of their epidemiology among the three genotypes of the BrYVs.

## References

[1] Xiang HY, Dong SW, Shang QX, Zhou CJ, Li DW, Yu JL, Han CG (2011). Molecular characterization of two genotypes of a new polerovirus infecting brassicas in China. Arch Virol.

[2] Lim S, Yoo RH, Igori D, Zhao F, Kim KH, Moon JS (2015). Genome sequence of a recombinant brassica yellows virus infecting Chinese cabbage. Arch Virol.

[3] Kamitani M, Nagano AJ, Honjo MN, Kudoh H. RNA-Seq reveals virus-virus and virus-plant interactions in nature. FEMS Microbiol Ecol. 2016;92. 10.1093/femsec/fiw176 PMC5854034 27549115

[4] Zhang XY, Xiang HY, Zhou CJ, Li DW, Yu JL, Han CG (2014). Complete genome sequence analysis identifies a new genotype of brassica yellows virus that infects cabbage and radish in China. Arch Virol.

[5] Zhang XY, Dong SW, Xiang HY, Chen XR, Li DW, Yu JL, Han CG (2015). Development of three full-length infectious cDNA clones of distinct brassica yellows virus genotypes for agrobacterium-mediated inoculation. Virus Res.

[6] Xiang HY, Shang QX, Han CG, Li DW, Yu JL (2008). Complete sequence analysis reveals two distinct poleroviruses infecting cucurbits in China. Arch Virol.

[7] Hammond R, Crosslin J, Pasini R, Howell W, Mink G (1999). Differentiation of closely related but biologically distinct cherry isolates of Prunus necrotic ringspot virus by polymerase chain reaction. J Virol Methods.

[8] Nie XZ, Singh RP (2000). Detection of multiple potato viruses using an oligo (dT) as a common cDNA primer in multiplex RT-PCR. J Virol Methods.

[9] Nie XZ, Singh RP (2001). A novel usage of random primers for multiplex RT-PCR detection of virus and viroid in aphids, leaves, and tubers. J Virol Methods.

[10] Bertolini E, Olmos A, López MM, Cambra M (2003). Multiplex nested reverse transcription-polymerase chain reaction in a single tube for sensitive and simultaneous detection of four RNA viruses and Pseudomonas savastanoi pv. savastanoi in olive trees. Phytopathology.

[11] Ratti C, Clover GR, Autonell CR, Harju VA, Henry CM (2005). A multiplex RT-PCR assay capable of distinguishing beet necrotic yellow vein virus types A and B. J Virol Methods.

[12] Gambino G, Gribaudo I (2006). Simultaneous detection of nine grapevine viruses by multiplex reverse transcription-polymerase chain reaction with coamplification of a plant RNA as internal control. Phytopathology.

[13] Deb M, Anderson JM (2008). Development of a multiplexed PCR detection method for Barley and Cereal yellow dwarf viruses, Wheat spindle streak virus, Wheat streak mosaic virus and Soil-borne wheat mosaic virus. J Virol Methods.

[14] Wei T, Lu G, Clover G (2009). A multiplex RT-PCR for the detection of Potato yellow vein virus, Tobacco rattle virus and Tomato infectious chlorosis virus in potato with a plant internal amplification control. Plant Pathol.

[15] Wintermantel WM, Hladky LL (2010). Methods for detection and differentiation of existing and new crinivirus species through multiplex and degenerate primer RT-PCR. J Virol Methods.

[16] Yokomi R, Saponari M, Sieburth P (2010). Rapid differentiation and identification of potential severe strains of Citrus tristeza virus by real-time reverse transcription-polymerase chain reaction assays. Phytopathology.

[17] Chen SN, Gu H, Wang XM, Chen JS, Zhu WM. Multiplex RT-PCR detection of Cucumber mosaic virus subgroups and Tobamoviruses infecting Tomato using 18S rRNA as an internal control. Acta Biochim Biophys Sin. 2011: gmr031. 10.1093/abbs/gmr031 21531735

[18] Abarshi M, Mohammed I, Jeremiah S, Legg J, Kumar PL, Hillocks R, Maruthi M (2012). Multiplex RT-PCR assays for the simultaneous detection of both RNA and DNA viruses infecting cassava and the common occurrence of mixed infections by two cassava brown streak viruses in East Africa. J Virol Methods.

[19] Dai J, Cheng JL, Huang T, Zheng X, Wu YF (2012). A multiplex reverse transcription PCR assay for simultaneous detection of five tobacco viruses in tobacco plants. J Virol Methods.

[20] Panno S, Davino S, Rubio L, Rangel E, Davino M, García-Hernández J, Olmos A (2012). Simultaneous detection of the seven main tomato-infecting RNA viruses by two multiplex reverse transcription polymerase chain reactions. J Virol Methods.

[21] Cho S, Jeong R, Yoon Y, Lee S, Shin D, Kang H, Lee B (2013). One-step multiplex reverse transcription-polymerase chain reaction for the simultaneous detection of three rice viruses. J Virol Methods.

[22] Kwon JY, Ryu KH, Choi SH (2013). Reverse transcription polymerase chain reaction-based system for simultaneous detection of multiple lily-infecting viruses. Plant Pathol J.

[23] Noorani MS, Awasthi P, Sharma MP, Ram R, Zaidi AA, Hallan V (2013). Simultaneous detection and identification of four cherry viruses by two step multiplex RT-PCR with an internal control of plant nad5 mRNA. J Virol Methods.

[24] Yu Y, Zhao Z, Jiang D, Wu Z, Li S (2013). A one-step multiplex RT-PCR assay for simultaneous detection of four viruses that infect peach. Lett Appl Microbiol.

[25] Ali RN, Dann AL, Cross PA, Wilson CR (2014). Multiplex RT-PCR detection of three common viruses infecting orchids. Arch Virol.

[26] Zhao XT, Liu XL, Ge BB, Li MJ, Hong B. A multiplex RT-PCR for simultaneous detection and identification of five viruses and two viroids infecting chrysanthemum. Arch Virol. 2015;1–8. 10.1007/s00705-015-2360-z 25698104

[27] Saade M, Aparicio F, Sanchez-Navarro J, Herranz M, Myrta A, Di Terlizzi B, Pallas V (2000). Simultaneous detection of the three ilarviruses affecting stone fruit trees by nonisotopic molecular hybridization and multiplex reverse-transcription polymerase chain reaction. Phytopathology.

[28] Uga H, Tsuda S (2005). A one-step reverse transcription-polymerase chain reaction system for the simultaneous detection and identification of multiple tospovirus infections. Phytopathology.

[29] Roy A, Ananthakrishnan G, Hartung JS, Brlansky R (2010). Development and application of a multiplex reverse-transcription polymerase chain reaction assay for screening a global collection of Citrus tristeza virus isolates. Phytopathology.

[30] Shang QX, Xiang HY, Li DW, Yu JL, Han CG (2012). Rapid detection and differentiation of three cucurbit-infecting poleroviruses by multiplex RT–PCR. J Agric Sci.

[31] Panno S, Ferriol I, Rangel EA, Olmos A, Han C-G, Martinelli F, Rubio L, Davino S (2014). Detection and identification of Fabavirus species by one-step RT-PCR and multiplex RT-PCR. J Virol Methods.

[32] Han CG, Li DW, Xing YM, Zhu K, Tian ZF, Cai ZN, Yu JL, Liu Y (2000). Wheat yellow mosaic virus widely occurring in wheat (*Triticum aestivum*) in China. Plant Dis.

[33] Elnifro EM, Ashshi AM, Cooper RJ, Klapper PE (2000). Multiplex PCR: optimization and application in diagnostic virology. Clin Microbiol Rev.

[34] Markoulatos P, Siafakas N, Moncany M (2002). Multiplex polymerase chain reaction: a practical approach. J Clin Lab Anal.

[35] Bariana H, Shannon A, Chu P, Waterhouse PM (1994). Detection of five seedborne legume viruses in one sensitive multiplex polymerase chain reaction test. Phytopathology.

[36] Ito T, Ieki H, Ozaki K (2002). Simultaneous detection of six citrus viroids and Apple stem grooving virus from citrus plants by multiplex reverse transcription polymerase chain reaction. J Virol Methods.

[37] Sanchez-Navarro J, Aparicio F, Herranz M, Minafra A, Myrta A, Pallas V (2005). Simultaneous detection and identification of eight stone fruit viruses by one-step RT-PCR. Eur J Plant Pathol.

[38] Hauser S, Weber C, Vetter G, Stevens M, Beuve M, Lemaire O (2000). Improved detection and differentiation of poleroviruses infecting beet or rape by multiplex RT-PCR. J Virol Methods.

[39] Vigano F, Stevens M (2007). Development of a multiplex immunocapture-RT-PCR for simultaneous detection of BMYV and BChV in plants and single aphids. J Virol Methods.

[40] Yao BY, Wang GP, Ma XF, Liu WB, Tang HH, Zhu H, Hong N (2014). Simultaneous detection and differentiation of three viruses in pear plants by a multiplex RT-PCR. J Virol Methods.

[41] Song A, You Y, Chen F, Li P, Jiang J, Chen S (2013). A multiplex RT‐PCR for rapid and simultaneous detection of viruses and viroids in chrysanthemum. Lett Appl Microbiol.

[42] Tuo D, Shen W, Yang Y, Yan P, Li X, Zhou P (2014). Development and validation of a multiplex reverse transcription PCR assay for simultaneous detection of three papaya viruses. Viruses.

[43] Nie XZ, Singh RP (2002). A new approach for the simultaneous differentiation of biological and geographical strains of *Potato virus Y* by uniplex and multiplex RT-PCR. J Virol Methods.

[44] Nie XZ, Singh RP (2003). Specific differentiation of recombinant PVY N: O and PVY NTN isolates by multiplex RT-PCR. J Virol Methods.

[45] Crosslin J, Hamm P, Shiel P, Hane D, Brown C, Berger P (2005). Serological and molecular detection of tobacco veinal necrosis isolates of Potato virus Y (PVY N) from potatoes grown in the Western United States. Am J Potato Res.

[46] Lorenzen JH, Piche LM, Gudmestad NC, Meacham T, Shiel P (2006). A multiplex PCR assay to characterize potato virus Y isolates and identify strain mixtures. Plant Dis.

[47] Liu XP, Lu WC, Liu YK, Li JL (1990). A study of TuMV strain differentiation of cruciferous vegetables from 10 provinces in China-new host differentiator screening and strain classification. Chin Sci Bull.

[48] Wang S, Li L, Fang X, Huang Z (1990). Surveys of rapeseed virus diseases in the southern China and serological diagnosis. Plant Prot.

[49] Francki R, Mossop D, Hatta T. Cucumber mosaic virus. CMI/AAB descriptions of plant viruses. 1979;213(6).

